# Trial and Failure of Naphtha

**Published:** 1844-03-23

**Authors:** Wm. H. Ranking

**Affiliations:** Cantab.


TRIAL AND FAILURE OF NAPHTHA.

BY WM. H. RANKING, M. D., CANTAB.

When any particular medicine is vaunted for its success in the treatment of a disease usually considered to be incurable, it is but right that both sides of the question should be viewed with equal attention. As a *per contra*, therefore, to the two cases published in the Lancet (pages 216, 251,) as corroborative of the reality of Dr. Hastings' specific for consumption, I think it my duty to bring forward some instances of the utter failure of the medicine, (naphtha). In doing so I do not think it necessary to apologise for the empiricism of the proceeding, as I look upon confirmed phthisis as one of the very few diseases in which such practice can be justified. It is true that the cases in which I have hitherto employed naphtha were not very promising, but it was my determination, in testing the powers of the remedy, to exhibit it only in cases concerning the nature of which there could be no doubt. I have given it in eight cases—three females and five males. Of these, three are already dead, the remainder are still alive, but dying gradually. In five of these cases there were unequivocal signs of cavities in one or both lungs, with emaciation and copious purulent expectoration. In the other three the disease was not so far advanced; but besides the signs of tubercular deposit, crepitous and submucous râles, with the appearance of pus in the sputa, indicated that softening had commenced. In none of these cases could I perceive the most trivial benefit from the medicine; neither was the perspiration checked, as it is stated to be, as if by magic; nor were the cough and expectoration diminished. In two instances nausea was complained of, and every patient, without exception, implored its omission. I mention these cases, not to discourage others from trying naphtha, for I should, in common with every humane man, be happy to think that a check was at last found for so fearful a scourge as phthisis, but to guard against a too confident reliance upon its powers. With regard to the cases published in the Lancet, Mr. Wilson will, perhaps, excuse my saying that his case is not an instance which can be taken as an unequivocal testimony in favour of naphtha. For, granting that there was tubercular disease at all, which the remark of one of the two "acknowledged stethoscopists" ("I *think* there is obstruction in the left lung, most probably crude tubercles") leaves somewhat doubtful, there are, at all events, no symptoms but those of the most incipient stage of the disease. The sound, on percussion, not decidedly dull, but only "*duller*" over the right infraclavicular space than over the left side, the intermitting cough without expectoration, indicate no more than the existence of scattered tubercles. Now this is a condition which is occasionally removed and more frequently suspended by the usual modes of treatment. No stress, therefore, can be laid upon the cure of such a case by naphtha. But if the disease has only been suspended by its use, and there is no evidence of its entire removal, the value of the case is still further diminished.

The other case (Lancet, p. 216) is worthless, for two reasons. In the first place, it is called phthisis in the Lancet, and chronic bronchitis in the Medical Gazette; and, in the second place, one portion of the narrative is flatly denied (Lancet, p. 80) by the physicians of the Colchester Hospital; so that a certain degree of doubt involves the whole. In conclusion, I repeat, that I have no object in this communication but to set the subject in a true light. The medicine is, perhaps, worthy of future trial, and such I intend to give it.—*Lon. Lancet*, Dec., 1844.

